# Lysine or histidine? examining the role of the proximal ligand at the active heme center of cytochrome *c* nitrite reductase

**DOI:** 10.1007/s00775-026-02165-w

**Published:** 2026-07-28

**Authors:** Bianca Hermann, Marc Rudolf, Albrecht Messerschmidt, Jörg Simon, Oliver Einsle, Peter M.H. Kroneck

**Affiliations:** 1 Institute for Physical Chemistry, Albert-Ludwigs-University Freiburg, Albertstrasse 21, 79104 Freiburg, Germany; 2 Department of Biology, University of Konstanz, Universitätsstrasse 10, 78457 Konstanz, Germany; 3 Abteilung Strukturforschung, Max-Planck-Institut für Biochemie, Am Klopferspitz 18a, 82152 Martinsried, Germany; 4 Microbial Energy Conversion and Biotechnology, Department of Biology, Technical University of Darmstadt, Schnittspahnstraße 10, 64287 Darmstadt, Germany; 5 Institute for Biochemistry, Albert-Ludwigs-University Freiburg, Albertstrasse 21, 79104 Freiburg, Germany

**Keywords:** *Campylobacter jejuni*, Cytochrome *c* nitrite reductase NrfA, Dissimilatory nitrate reduction to ammonium, Multiheme cytochrome *c*, Nitrogen cycle, *Wolinella succinogenes*

## Abstract

The identity of the proximal ligand of its catalytic heme 1 center - lysine or histidine - may directly affect how efficiently pentaheme cytochrome *c* nitrite reductase catalyzes the six-electron reduction of nitrite (NO_2_^−^) to ammonium (NH_4_^+^) and may also alter substrate specificity. This reaction is part of the pathway of dissimilatory nitrate reduction to ammonium, which competes with denitrification and avoids the formation of unreactive dinitrogen (N_2_). In this study, we compare the biochemical properties and structures of cytochrome *c* nitrite reductase from *Campylobacter jejuni* with those of the enzyme from *Wolinella succinogenes* and examine its Lys134His variant. Notably, both the *Campylobacter* nitrite reductase and the Lys134His variant possess a catalytic heme 1 center coordinated by histidine as the proximal ligand, whereas the native *Wolinella* enzyme is coordinated by lysine. Despite this difference, both enzymes catalyze the reduction of NO_2_^−^ to NH_4_^+^ with high specific activities. To enable biochemical and structural characterization of these enzymes, we developed optimized protocols for protein expression and purification. The data we present allow, for the first time, a direct analysis of the influence of histidine or lysine as the proximal nitrogen ligand at the catalytic heme 1 center of pentaheme cytochrome *c* nitrite reductase within otherwise similar protein backgrounds.

**Supplementary Information:**

The online version contains supplementary material available at https://doi.org/10.1007/s00775-026-02165-w.

## Introduction

The enzyme studied in this work, pentaheme cytochrome *c* nitrite reductase (NrfA; for nitrite reduction by formate), is a key player in microbial nitrogen metabolism [1–3]. NrfA reduces nitrite to ammonium in a single step with excellent activity (Eq. 1), as part of the dissimilatory nitrate reduction to ammonium process (DNRA; ^+V^NO_3_^−^→ ^+III^NO_2_^−^→^−III^NH_4_^+^) [4–6]. Electron and proton delivery to the substrate nitrite ensures that both N–O bonds are cleaved, and no detectable intermediates are released. NrfA chemistry is especially remarkable, given that the alternative pathway, denitrification plus nitrogen fixation, requires four highly complex metalloenzymes and generates nitric oxide (NO), nitrous oxide (N_2_O), and dinitrogen (N_2_) as separate intermediates [7–9]. NrfA also converts NO and NH_2_OH to NH_4_^+^, and SO_3_^2−^ to H_2_S at considerable rates, thus forming a link between the biogeochemical cycles of nitrogen and of sulfur (Eq. 2) [10–15].

1 $$^{{\rm{ + III}}}{\rm{N}}{{\rm{O}}_{\rm{2}}}^{\rm{ - }}{\rm{ + 6}}{{\rm{e}}^{\rm{ - }}}{\rm{ + 8}}{{\rm{H}}^{\rm{ + }}}\rightarrow{{\rm{}}^{{\rm{ - III}}}}{\rm{N}}{{\rm{H}}_{\rm{4}}}^{\rm{ + }}{\rm{ + 2}}{{\rm{H}}_{\rm{2}}}{\rm{O\;\;\;\;(}}{{\rm{E}}^{\rm{,}}}_{\rm{o}}{\rm{ = + 0}}{\rm{.340 V)}}$$

2 $$^{+\mathrm{IV}}\mathrm{SO}_3^{2-} + 6\mathrm{e}^- + 8\mathrm{H}^+ \rightarrow \mathrm{H}_2^{-\mathrm{II}}\mathrm{S} + 3\mathrm{H}_2\mathrm{O} \quad (\mathrm{E}_\circ = -0.117\mathrm{V})$$

Another remarkable feature of multiheme NrfA enzymes is the unusual C-X_1_-X_2_-C-K heme-binding motif. Cole and co-workers first identified this unique motif through sequence analysis of NrfA from *Escherichia coli* [16]. Einsle and colleagues confirmed the C-X_1_-X_2_-C-K motif by X-ray crystallography in NrfA structures from the Epsilon-proteobacteria *Sulfurospirillum deleyianum* (SdNrfA, PDB 1QD8) [17] and *Wolinella succinogenes* (WsNrfA, PDB 1FS7) [18]. The novel heme-binding motif was suggested to be crucial for promoting anion binding and providing a suitable reduction potential for the NO_2_^−^→NH_4_^+^ reaction [19, 20]. The motif provides a lysine (amino nitrogen, N_sp3_) as the proximal ligand of the active-site heme 1. Three conserved residues — His, Tyr, and Arg — define the distal pocket of the active site. A Ca^2+^ ion is bound approximately 11 Å from the active-site heme iron. The positive electrostatic potential from this ion helps to attract NO_2_^−^ to the active site. The Ca^2+^ ion also likely facilitates proton transfer to the substrate and catalytic intermediates by stabilizing water molecules or helping in proton relay. Four low-spin *c*-type hemes are bound through C-X_1_-X_2_-C-H heme-binding motifs. These hemes deliver electrons to the active-site heme 1, which is high-spin in the absence of substrate or inhibitor. The low-spin hemes have His/His axial ligation and are arranged as two branches converging on the active site from different regions of the protein surface. One branch supports electron exchange between the active site and the cellular redox partner. The second branch enables electron exchange between NrfA subunits in the homodimeric structures [20]. The basic structural features, heme packing, and anatomy of the catalytic heme 1 cavity were also found in other NrfA structures (*Escherichia coli*; PDB 2RDZ [21], *Shewanella oneidensis*; PDB 3UBR [22], *Desulfovibrio desulfuricans*; PDB 1OAH [23], and *Desulfovibrio vulgaris*; PDB 2J7A [24]. A structural variation close to heme 1 was reported for *Geobacter lovleyi* NrfA (PDB 6V0A). The space filled by Ca²⁺ in canonical NrfA enzymes was occupied by an arginine residue in GlNrfA [25]. A second Ca²⁺-independent NrfA protein, NrfA-1, was discovered in *Geobacter metallireducens* (PDB 8BCX), and the structure showed an altered active site [26].

Recent studies have enhanced the understanding of NrfA nitrite reductase diversity and phylogeny through analyses of full-length protein sequences. Several clades possess the C-X_1_-X_2_-C-H motif in the catalytic heme-1 binding domain, rather than the C-X_1_-X_2_-C-K motif [27–29]. This observation is consistent with research on *Campylobacter jejuni*, which demonstrates that the putative *nrfA* gene contains five C-X_1_-X_2_-C-H heme binding motifs, indicating a histidine (imidazole nitrogen, N_sp2_) as the proximal ligand of the active-site heme 1 [30–32]. Accordingly, the present study compares the structures and properties of *C. jejuni* cytochrome *c* nitrite reductase (CjNrfA) and the variant WsNrfA-Lys134His, using wild-type WsNrfA as a reference. Optimized protocols for expression and purification have been developed, producing substantial quantities of pure protein and facilitating comparative characterization of CjNrfA and the WsNrfA-Lys134His variant [33–35]. Both proteins efficiently catalyze the reduction of NO_2_^−^ to NH_4_^+^, as well as the reduction of SO_3_^2−^ to H_2_S. This methodology enables, for the first time, a direct investigation of the effect of either a histidine or a lysine residue as the proximal nitrogen ligand at the catalytic heme 1 center of NrfA in otherwise comparable protein backgrounds.

## Results

### *Campylobacter jejeuni* cytochrome *c* nitrite reductase

#### Activity and protein architecture

**Fig. 1:**
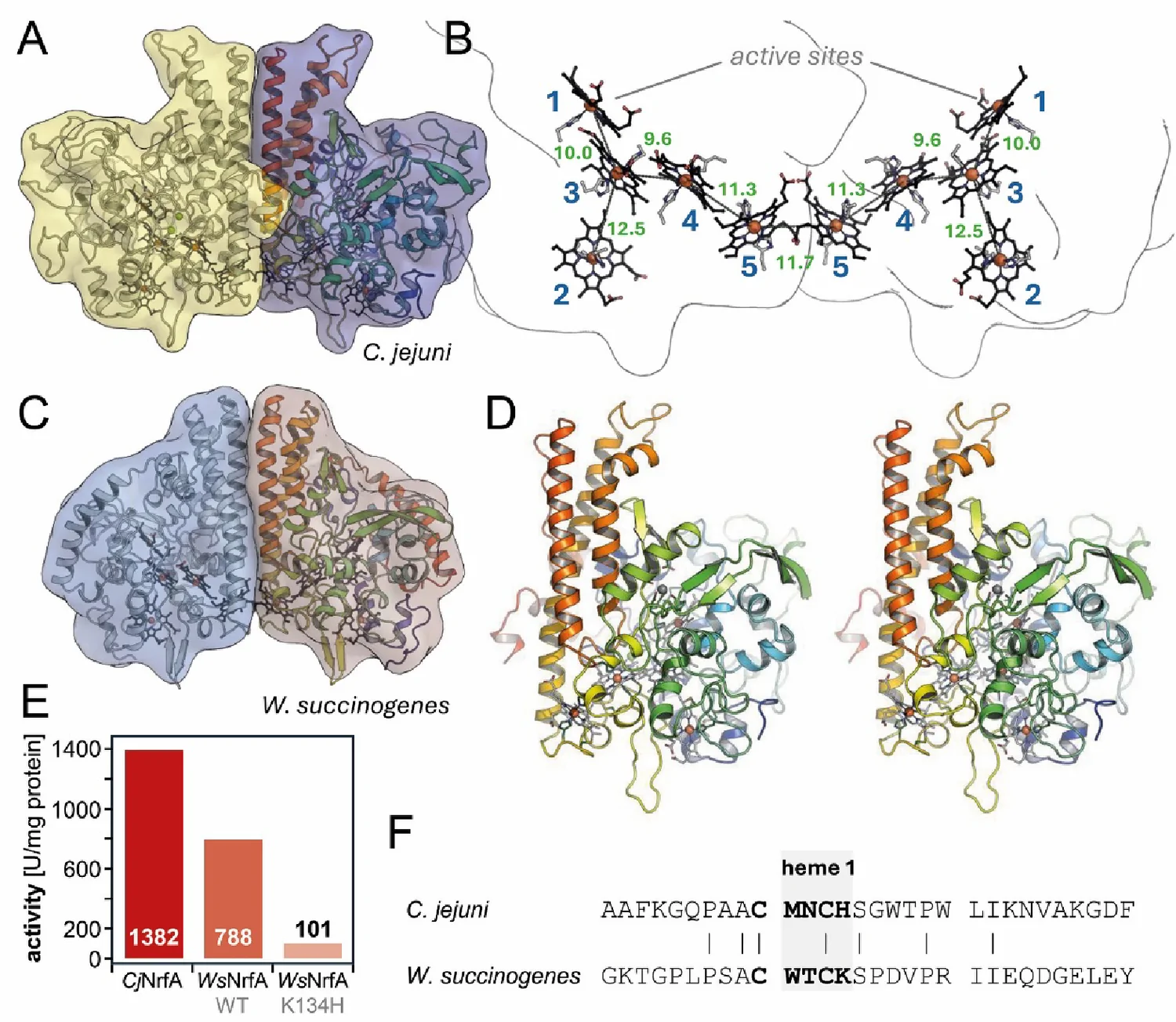
Cytochrome *c* nitrite reductases from *C. jejuni* and *W. succinogenes*. (**A**) Dimer structure of CjNrfA, with the characteristic, C-terminal helices forming the dimer interface. (**B**) The heme groups are arranged in the characteristic pentaheme motif, with Fe-Fe distances of between 9.6 and 12.5 Å, and 11.7 Å between hemes 5 at the dimerization interface enabling fast electron transfer within and between subunits of the NrfA dimer; heme 1 is the active site, heme 2 the likely electron entry point from tetraheme cytochrome *c* NrfH. (**C**) Dimer of WsNrfA. (**D**) Stereo representation of the CjNrfA monomer, coloured from blue at the N-terminus to red at the C-terminus. (**E**) Specific nitrite reductase activity (BV_red_ →NO_2_^−^) of CjNrfA, WsNrfA, and WsNrfA-Lys134His. (**F**) Sequences surrounding the heme 1 binding motif C-M-N-C-H (CjNrfA) and C-W-T-C-K (WsNrfA)

CjNrfA was produced in *W. succinogenes* cells and purified to homogeneity by affinity chromatography as described in the Methods section. The protein migrated as a single band (SDS PAGE), with a relative molecular mass of 73 kDa. The sample, showing a single tetramer band on BN-PAGE, was analyzed by size exclusion chromatography (Supplementary File, Figs. 1 and 2). The relative molecular mass of the eluted sample was determined to be 137.6 kDa by static light scattering, corresponding to a dimer. The tetrameric form was therefore interpreted as an effect of sample concentration (> 12 mg mL^− 1^), with no physiological relevance. In the oxidized (as isolated) or in the reduced (dithionite; photochemically with deazaflavin) state the protein displayed the characteristic cytochrome *c* UV/vis absorption and X-band EPR spectra [36–38]. CjNrfA converts NO_2_^−^ to NH_4_^+^ with a higher nitrite reductase activity (1382 U mg^− 1^ protein/100%) compared to wild-type WsNrfA (788/57%), and the variant WsNrfA-Lys134His (101/7.3%) (Fig. 1); possible intermediates NO and H_2_NOH are also converted to NH_4_^+^, as well as SO_3_^2−^ to H_2_S (data not shown).

The overall architecture of CjNrfA (1.6 Å resolution) (Fig. 1) is very similar to that reported for wild-type WsNrfA (1.6 Å, PDB 1FS7), carrying a compact, heme-containing N-terminal globular domain and three long α-helices on the C-terminal side that form the characteristic dimer interface [18]. The CjNrfA protein (2 × 73 kDa) is larger than the WsNrfA protein (2 × 58 kDa), or other NrfA proteins described so far [20]. This does not affect the central core structure including the catalytic heme 1 cavity and the residual four heme groups but has an impact on the structure of the C-terminal domain. The monomer can be subdivided into an N-terminal globular domain, harboring all five heme groups, and a C-terminal part mainly built up by the three long α-helices that form the dimerization interface through hydrophobic interactions. The C-terminus consists of a long loop with three short helices running alongside the globular domain. The α-helix 27 blocks the outlet of the otherwise conserved substrate/product channel structure. There are three distinct Ca^2+^ sites in each CjNrfA monomer, compared to one in the wild-type WsNrfA monomer, as discussed below. All hemes except the catalytic heme 1 are bis-His ligated and linked to the peptide backbone by thioether bonds to the cysteine residues of the classical heme-binding motif C-X_1_-X_2_-C-H. The porphyrin rings show a slight distortion from planarity, most strongly in heme 2 and least in the catalytic heme 1.

### Active-site cavity, substrate channel, and substrate binding

**Fig. 2:**
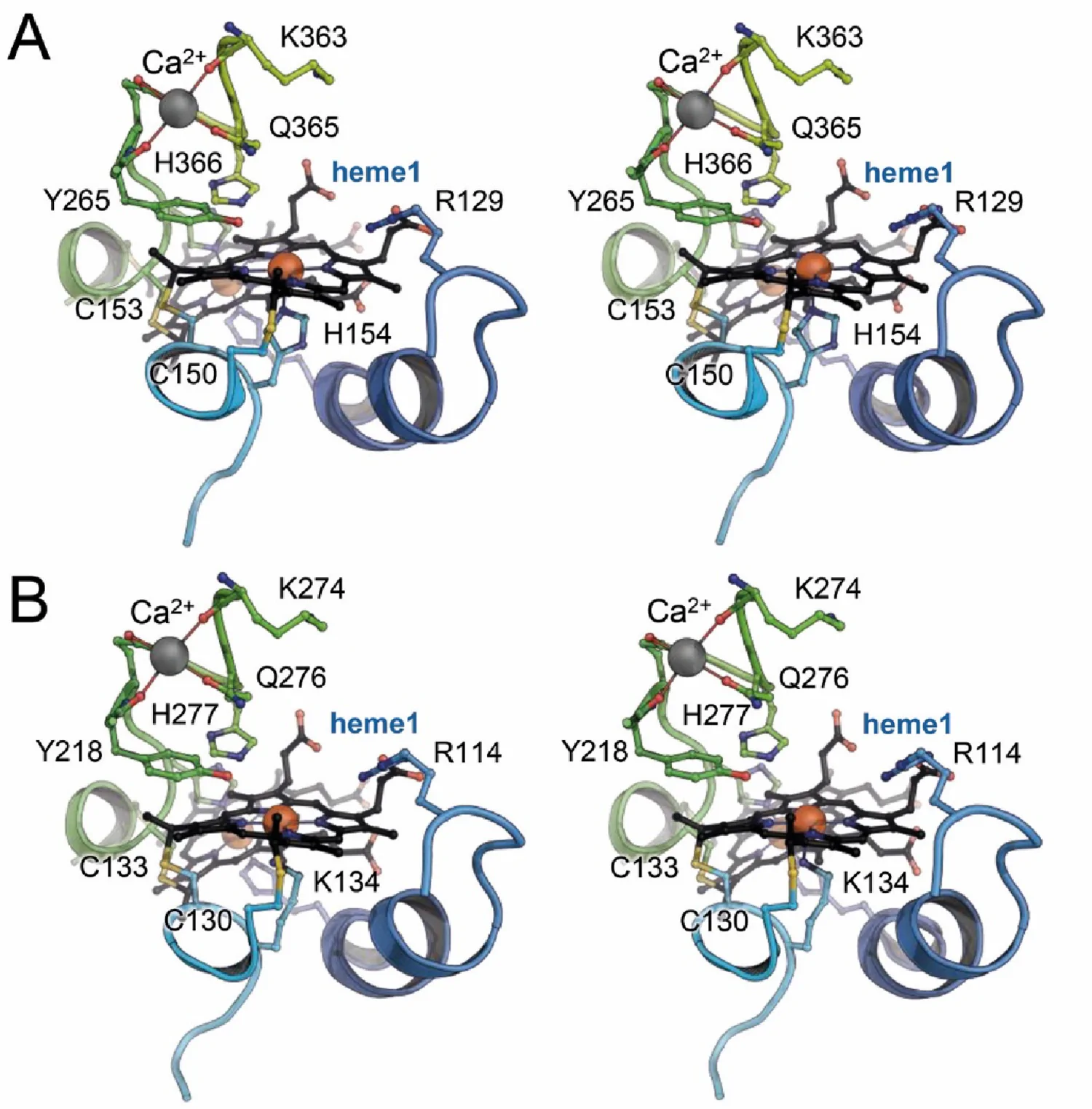
Stereo view of the active-site heme 1 of CjNrfA (**A**) and wild-type WsNrfA (**B**); Fe red, Ca^2+^ I grey; Fe is coordinated on the proximal side by imidazole N_sp2_ of His154 in CjNrfA, and by amino N_sp3_ of Lys134 in WsNrfA

**Fig. 3:**
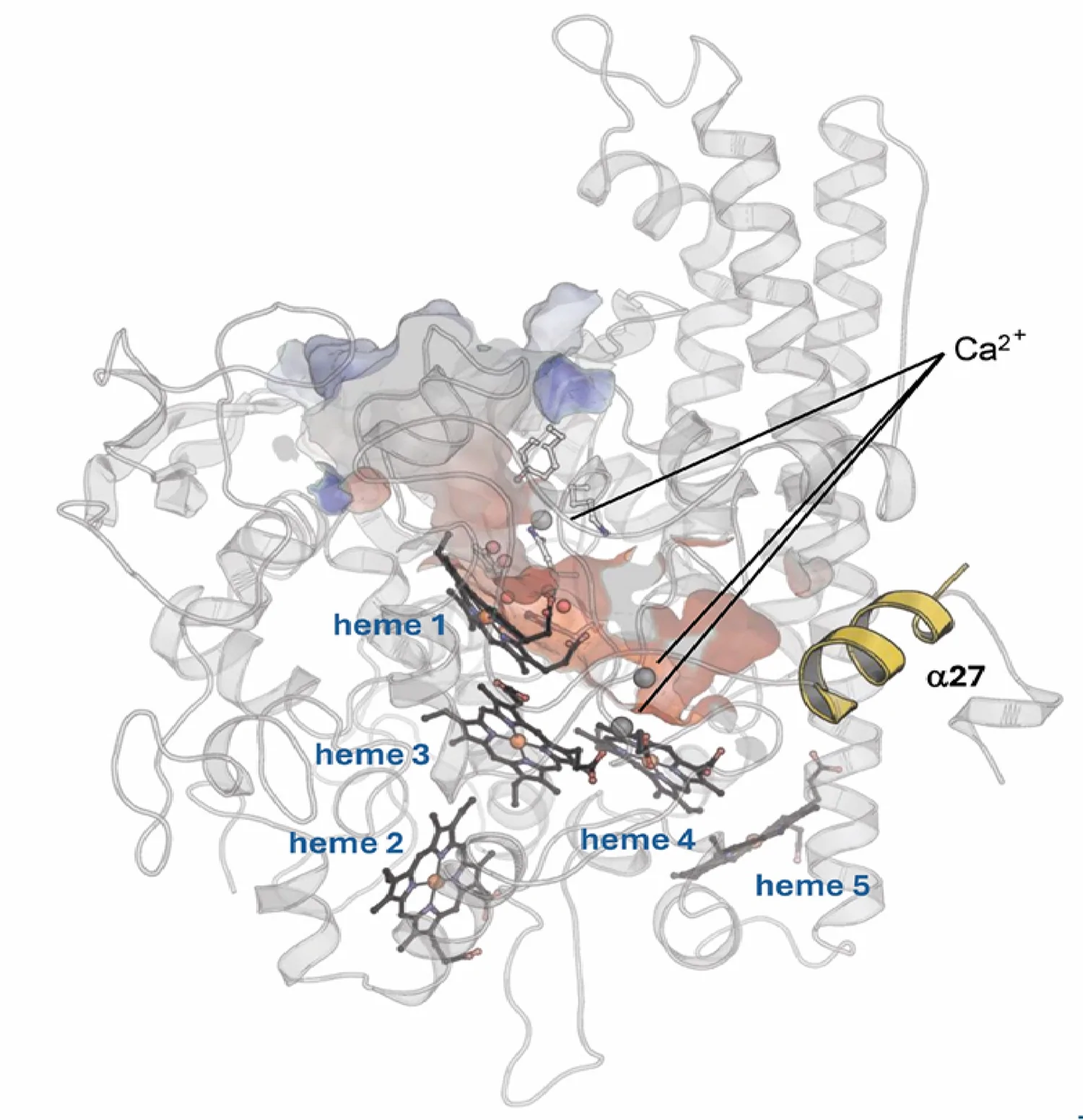
View of the CjNrfA substrate channel and the three calcium sites. The channel architecture is conserved, it passes the active site heme 1 and changes the electrostatic surface potential from positive (blue, left) near the entrance for anions (NO_2_^−^) to negative (red); the putative product exit observed in wild-type WsNrfA, is blocked by helix α 27 of the C-terminus; Ca II and Ca III (distance 4.1 Å) are located between heme 3 and heme 4, and both coordinated by oxygen ligands

The basic features of the distal side of active-site heme 1 detected so far in all NrfA crystal structures, i.e., the catalytic triad Arg, Tyr, His, as well as the Ca^2+^ ion, are conserved in the CjNrfA structure (Fig. 2). On the proximal side, Fe is coordinated by the imidazole nitrogen (N_sp2_) of His154, with a Fe-N distance of 2.26 Å, corresponding to a regular distance observed for heme His ligation. The distal side of the active-site cavity is lined by the catalytic residues Arg129, Tyr265, and His366, and the calcium-ligating residues Glu264 and Gln365. A water molecule is bound to the active site heme 1 iron. The 7-coordinate Ca^2+^ ion is positioned 10.4 Å above the heme 1 iron. Besides the Glu264 and Gln365 oxygen atoms its coordination sphere is completed by the backbone carbonyls of Tyr265 and Lys363, as well as two H_2_O molecules, as is the case in WsNrfA [18]. Two additional binding sites for divalent cations are defined in the CjNrfA structure between heme 3 and heme 4, most probably occupied by Ca^2+^ ions in the native state of CjNrfA (Fig. 3). The channel architecture is conserved, it passes the active site heme 1 and changes the electrostatic surface potential from positive (blue, left) near the entrance for anions (NO_2_^−^) to negative (red); the putative product exit observed in wild-type WsNrfA, is blocked by helix α27 of the C-terminus. In the substrate-bound structures, the electron density indicates that the Ca^2+^ II and Ca^2+^ III are partially replaced by Sr^2+^ ions as SrCl_2_ was an additive in the crystallization buffer. Apart from the proximal His ligation, the overall NrfA active site anatomy is highly conserved. This also holds for the binding mode of the native substrate NO_2_^−^, ligated by His366 and Arg129 (distances 2.3Å and 3.1Å, respectively), or SO_3_^2−^, ligated by the three catalytic residues Arg129, Tyr265, and His366 (distances 3.2Å, 2.7Å, and 2.5Å) (Supplementary File, Fig. 3).

### *Wolinella succinogenes* cytochrome *c* nitrite reductase Lys134His variant

#### Activity, protein architecture, and active-site cavity

**Fig. 4:**
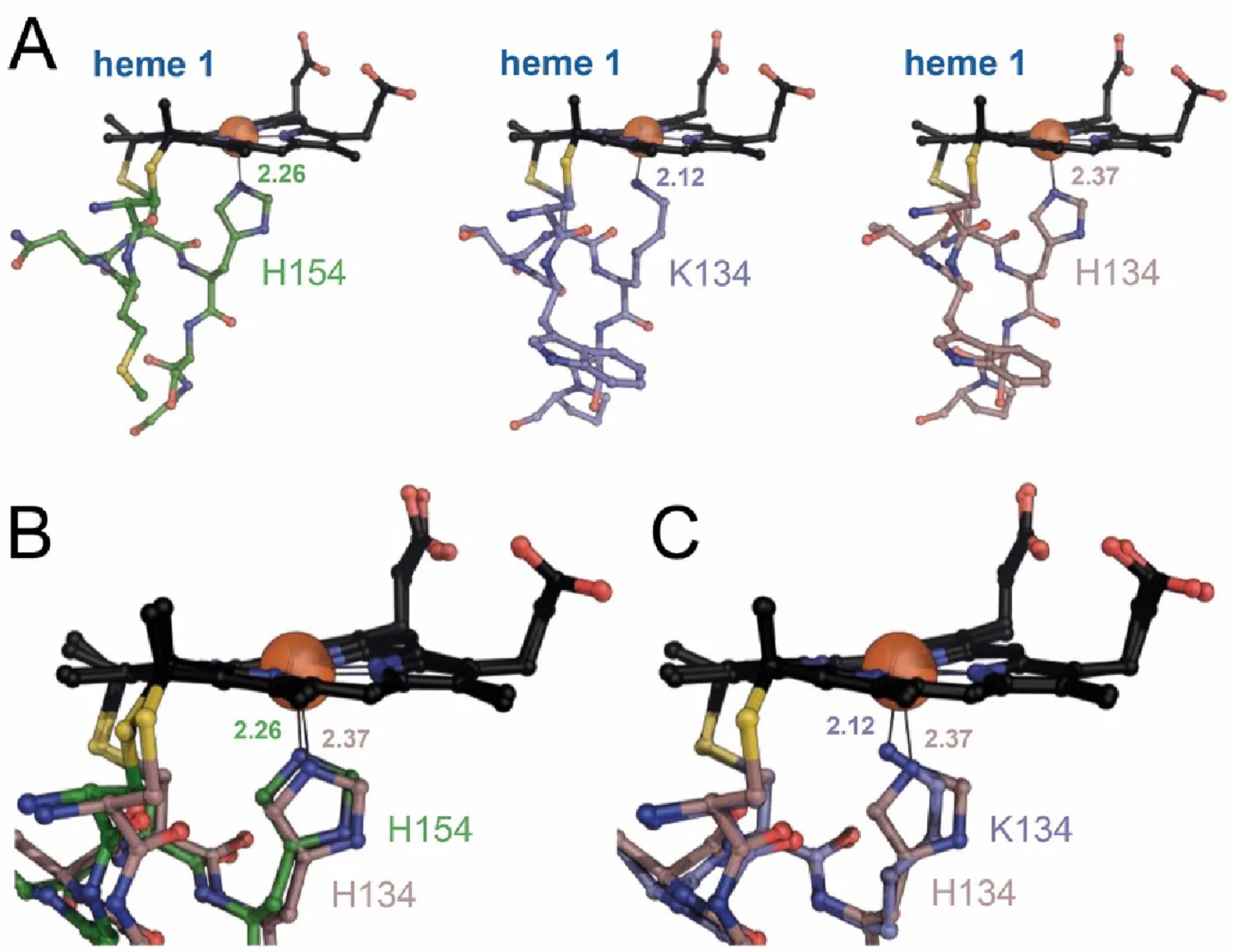
Comparative view of active-site heme 1 of CjNrfA (Fe-N_His154_ 2.26 Å), WsNrfA (Fe-N_Lys134_ 2.12 Å), and variant WsNrfA-Lys134His (Fe-N_His134_ 2.37 Å) (**A**); overlay of heme 1 proximal nitrogen ligands His154 (CjNrfA) and His134 (WsNrfA variant) (**B**), and of Lys134 (WsNrfA) and His134 (WsNrfA variant (**C**)

WsNrfA-Lys134His variant cells were prepared as described [33]. The protein was purified from the membrane fraction [37] as a homodimer (single band on SDS PAGE, relative molecular mass 58 kDa). Spectroscopic properties (UV/Vis, EPR) of the variant protein in the oxidized and reduced state are basically identical to those of wild-type WsNrfA [36–38]. WsNrfA-Lys134His converts NO_2_^−^ to NH_4_^+^, however with a lower activity compared to CjNrfA and WsNrfA (Fig. 1). The specific nitrite reductase activity of the mutant strain K134H, and the cell homogenate, did not exceed 40% of that of the wild-type. Note that strain K134H did not grow with nitrite as electron acceptor and its electron transport activity from formate to nitrite was about 5% of the wild-type activity [33]. The overall structure of the protein (1.9 Å resolution) reveals the characteristic dimer architecture, heme arrangement, substrate entrance and product exit channels, the primary active site residues Tyr218, His277, and Arg114, and the calcium site. All these key features are practically identical to those of wild-type WsNrfA (1.6 Å, PDB 1FS7). The protein scaffold seems to accommodate the Lys-His mutation, however, the Fe-N_His_ coordination requires some movement of heme 1 within the binding cavity (Fig. 4). The Fe-N_His_ distance is 2.37 Å, thus significantly longer than Fe-N_Lys_ (2.12 Å) in wild-type WsNrfA and the Fe-N_His_ distances of ≈ 2 Å of the bis-His coordinated hemes 2–5. The side chain of His134 is not as flexible as that of Lys134 to move the imidazole N_ε2_ nitrogen closer to the heme 1 iron to allow for optimal coordination with an angle of 90^o^, thereby achieving a shorter Fe-N_His_ bond distance as observed in CjNrfA (2.26 Å). The conformation of the heme 1 porphyrin ring appears to be less ruffled in WsNrfA-Lys134His than in wild-type WsNrfA, the position of the Fe atoms differs by ≈ 0.13 Å. The movement of Fe towards the proximal histidine ligand is prevented by the stacking interaction between heme 1 and heme 3, and the covalent fixation of the porphyrin ring to the protein backbone via the thioether bridges (Cys130, Cys133). Finally, the bond angle C_δ2_-N_ε2_-Fe with 150.6° is larger than the angle of 120–130° observed in canonical Fe-His binding motifs. The overall structure of the calcium binding site, i.e. orientation of ligating residues Glu217, Gln276, Lys274, Tyr218, and position of Ca^2+^ cation is identical to that of wild-type WsNrfA. The exchange of the proximal ligand Lys134 by histidine does not induce significant changes of the shape of the substrate channel and the roof of the distal site of the catalytic cavity. The substrate binding side at heme 1 is occupied by acetate (CH_3_COO^−^, from crystallization buffer), in contrast to SO_4_^2−^ or H_2_O, in the wild-type WsNrfA structure [18]. The acetate anion is oxygen-bound, with hydrogen bonds to Tyr218, His277 and a water molecule that is hydrogen-bonded to Arg114 (data not shown).

## Discussion

In this study, we provide structural and functional data that enable direct analysis of how histidine (imidazole N_sp2_) acts as the proximal nitrogen ligand at the catalytic heme 1 center in pentaheme cytochrome *c* nitrite reductase (NrfA). We compare the nitrite reductase activity and important structural aspects, such as protein architecture, heme arrangement, active-site cavity, substrate channel, and binding, of NrfA from *Campylobacter jejuni* with those of NrfA from *Wolinella succinogenes*, which contains a lysine at the active-site heme 1, and the engineered Lys134His variant.

### Protein architecture and active-site cavity

The structure of CjNrfA illustrates a protein fold conserved across all reported NrfA structures, it aligns with WsNrfA (PDB1FS7) at a Cα-rmsd of 1.50 Å. Structural overlays and sequence alignments confirm that the deviation stems primarily from expanded loop regions, as detailed in Fig. 1. The core fold secures precise heme orientation for efficient electron transfer, corroborated by the rmsd of 0.68 Å in heme-only overlays. Two C-terminal fold variants are identified in NrfA structures. WsNrfA (PDB 1FS7) [18], EcNrfA (PDB 2RDZ) [21], SdNrfA (PDB 1QD8) [17], and SoNrfA (PDB 3UBR) [22] retain an elongated C-terminal helix; enzymes from *Desulfovibrio desulfuricans* (PDB 1OAH) [23] and *Desulfovibrio vulgaris* (PDB 2J7A) [24] display a largely unstructured loop with short helices. The CjNrfA structure mirrors the latter, with three brief helices spanning the globular domain, and α-helix 27 obstructing the outlet channel (Fig. 3).

The high specific activity of NrfA enzymes is driven by finely tuned channel structures and electrostatic potentials [17, 37]. Conserved residues create a positive electrostatic environment within the active site, favoring anion binding to the heme 1 iron and facilitating efficient substrate reduction (Supplementary File, Fig. 3) [12, 18]. Note that density functional theory supports key roles for conserved catalytic-cavity residues His and Arg, but not Tyr, in nitrite binding [39]. These residues store protons required for the reduction of NO_2_^−^ to NH_3_/NH_4_^+^, which are resupplied by water molecules. The geometry of the inlet channel leading to the active-site heme 1 is similar across all reported NrfA structures, whereas the outlet channel can vary significantly. In WsNrfA, EcNrfA, and SoNrfA, the inlet channel is positively charged, it crosses the positively charged activesite cavity and ends in a single negatively charged outlet channel. The change in the channel surface potential was reported as one of the driving forces of the reaction [18]. In SdNrfA, the outlet channel branches into two exit pathways, but it remains unclear whether the product uses both or prefers one. In DvNrfA and DdNrfA [23, 24], the exit is blocked by an additional loop. Therefore, the product must leave the active site either through the inlet channel or, during the reaction cycle, a conformational change must open the exit. This situation is also found in CjNrfA, with the exit channel blocked by C-terminal helix α 27 (Fig. 3). Comparison of nitrite and sulfite reductase activities (selectivity ratio) across different organisms shows that these ratios can vary. The rate of nitrite reduction is significantly lower in DdNrfA when compared with SdNrfA or EcNrfA, whereas the rate of sulfite reduction is highest in DdNrfA. The selectivity ratio for EcNrfA is 5550 vs. 658 for DdNrfA, indicating that EcNrfA shows specificity towards NO_2_^−^, while DdNrfA is less specific towards NO_2_^−^ reduction. As the active-site cavities of the NrfA enzymes are almost identical, differences in the observed rates of substrate reduction may be related to other structural features, including the active-site channels [19]. Their variations may reflect adaptations of the NrfA structures to different requirements across organisms.

### The proximal nitrogen ligand of active-site heme

Several NrfA enzymes, including CjNrfA studied here, utilize the canonical C-X_1_-X_2_-C-H motif. Despite this difference, nitrite reduction rates remain consistently high whether lysine or histidine coordinates the heme, and structural analysis reveals that the active-site anatomy of C-X_1_-X_2_-C-H enzymes closely matches that of C-X_1_-X_2_-C-K enzymes (Figs. 1 and 4) [20, 34, 35, 40]. Building on these findings, mutational studies reveal that converting a naturally lysine-ligated C-X_1_-X_2_-C-K NrfA into a histidine-ligated C-X_1_-X_2_-C-H disrupts the active-site geometry. The resulting Fe-N_His_ bond length becomes significantly elongated, suggesting that this simple substitution is insufficient for functional adaptation.

This observation raises questions about the role of the proximal heme 1 ligand.

In 2004, the octaheme cytochrome *c* tetrathionate reductase from *Shewanella oneidensis* was structurally characterized. Its catalytic heme iron center is ligated by proximal lysine, though it attaches via the canonical C-X_1_-X_2_-C-H motif. Here, histidine rotates away to allow coordination by lysine on a neighboring helix [41]. By contrast, in CjNrfA, the imidazole N_sp2_ atom (Fe-N_His_ 2.26 Ǻ) occupies nearly the same position as the amino N_sp3_ of lysine in WsNrfA (Fe-N_Lys_ 2.12 Ǻ). For the WsNrfA-Lys134His variant, the Fe-N_His_ distance is 2.37 Ǻ, and the imidazole ring adopts a slightly different orientation towards the iron, which causes a ruffling of the heme plane, a shift in iron position, and a significant decrease in activity (Figs. 1 and 4) [33].

The atomic-level catalytic mechanism has been described. Electronic changes lead to NO_2_^−^ activation and its reduction to NH_3_/NH_4_^+^, as inferred from density functional calculations and crystallographic data for WsNrfA intermediates [42]. The substrate binds to heme 1, *trans* to the proximal ligand Lys134. NO_2_^−^ reduction begins with heterolytic cleavage of one N–O bond, enabled by strong back-bonding interaction. This effect appears only in the reduced Fe(II) state and is key [42]. First calculations do not show any strong electronic reasons why nature would prefer an axial lysine, present in WsNrfA [18], over a conventional histidine ligand, present in CjNrfA [43–45]. Other naturally occurring heme proteins also exhibit lysine coordination. Prominent examples include the alkaline form of cytochrome *c* [46, 47], the hemoglobin THB1 of *Chlamydomonas reinhardtii* [48], the octaheme cytochrome *c* tetrathionate reductase of *Shewanella oneidensis* [41], and the hexameric octaheme cytochrome *c* nitrite reductase from *Thioalkalivibrio nitratireducens*, which hosts eight hemes *c* per subunit -seven *bis*-His ligated and one by the unique C-X_1_-X_2_-C-K motif [49]. Furthermore, the His93Gly sperm whale myoglobin cavity variant was engineered to probe ligand binding [50, 51]. Notably, substitution of proximal His93 with Lys resulted in an eightfold increase in the rate of NO_2_^−^→NO reduction in comparison to wild-type myoglobin [52].

Based on phylogenetic analysis, the C-X_1_-X_2_-C-K variant of NrfA likely arose first and was later replaced in some organisms by the C-X_1_-X_2_-C-H variant, as shown by structural and phylogenetic analysis [6, 53, 54]. For CjNrfA, two potential advantages of having the proximal Lys ligand at active-site heme 1 are apparent: (i) it may provide higher activity, and (ii) it could eliminate the need for a specialized cytochrome *c* synthase dedicated to the C-X_1_-X_2_-C-K motif, assuming *C. jejuni* ever possessed such an enzyme. Studies on *W. succinogenes* suggest that the C-X_1_-X_2_-C-K variant offers functional advantages over the C-X_1_-X_2_-C-H variant, based on analyses of mutant and protein variants [33–35].

Despite these evolutionary trends, the origins of lysine ligation remain speculative. It is possible that C-X_1_-X_2_-C-K NrfA enzymes either evolved from cytochrome systems with pre-existing lysine ligation, such as the octaheme nitrite reductase-type enzymes [49], or represent remnants of an ancestral, less specific cytochrome *c* biogenesis system that did not discriminate between lysine and histidine. Environmental pressures - such as nutrient composition, iron bioavailability, and redox conditions - might have reinforced either motif in different microbial lineages. Still, limited data on the diversity and physiology of nitrite-ammonifying clades prevent firm conclusions. Nonetheless, the adoption of the C-X_1_-X_2_-C-H motif among modern nitrite ammonifiers likely reflects an evolutionary optimization toward simplicity, efficiency, and ecological adaptability. The imidazole sidechain makes histidine perhaps the most versatile residue in proteins and enzymes. It is ubiquitously found at the active site of metalloenzymes. Its side chain bears a basic imidazole group, allowing it to play a vital role in intramolecular proton transfer under physiological conditions, and to act as a σ-donor and π-acceptor ligand, facilitating the formation of coordinate interactions with redox-active transition-metal ions [55].

### Outlook

Multiheme cytochromes *c* including NrfA continue to be of high interest in the bioremediation and bioenergy fields, in view of their capability to produce high current densities in microbial fuel cells. Deciphering their structural anatomy and mechanism of action has significantly advanced our understanding of the activation of inorganic nitrogen and sulfur compounds, multi-electron and multi-proton transfer, and extracellular redox reactions. Molecular dynamics simulations and quantum–mechanical/molecular-mechanical calculations will complement experiments and elucidate the choreography by which the cytochrome protein regulates the catalytic cycle [56–64].

## Methods

### Production and characterization of NrfA variant proteins

CjNrfA was produced in *W. succinogenes* cells as variants carrying a C-terminal Twin-Strep-tag [34, 35]; the protein was purified by affinity chromatography in a buffer containing 100 mM Tris/HCl, pH 8.0, 150 mM NaCl, and 2.5 mM d-desthiobiotin. Similarly, wild-type WsNrfA and the variant WsNrfA-Lys134His were purified from the membrane fraction [12, 33, 37]. Both CjNrfA and WsNrfA-Lys134His are homodimers, with each migrating on SDS PAGE as a single band, matching relative molecular masses of 73 kDa (CjNrfA) and 58 kDa (WsNrfA-Lys134His). In the oxidized (as isolated) state the proteins display the characteristic *c*-type cytochrome UV/vis absorption spectra, with intense absorption maxima at 280, 409 (Soret band), and 534 (WsNrfA-Lys134His)/531 (CjNrfA) nm, and the shoulder around 615 nm (WsNrfA-Lys134His), and at 620 nm (CjNrfA), respectively, that can be assigned to a high-spin Fe(III) heme center [36, 37]. Upon reduction with dithionite (Na_2_S_2_O_4_), or photochemically with deazaflavin/oxalate, the Soret maximum shifts to 419 nm, and new absorption maxima appear at 523 and 553 nm. The reduced proteins are fully reoxidized by turnover with nitrite, restoring the original absorption maxima of the proteins as isolated. EPR spectra of the as-isolated CjNrfA and the WsNrfA-Lys134His variant, recorded at X-band (≈ 9.50 GHz, perpendicular mode, 5–10 K; ≈ 9.1 GHz, parallel mode, 5 K), reveal the complex pattern of resonances reported earlier for several NrfA enzymes, e.g., SdNrfA, WsNrfA, EcNrfA, and DsNrfA, assigned to the active site heme 1 (high-spin Fe(III) with Lys and H_2_O as axial ligands, and to hemes 2 to 5 (low-spin Fe(III) with His-His ligation) [21, 38, 65–68].

### Nitrite reductase activity

Nitrite reductase activity was measured photometrically under anoxic conditions at 37 °C by recording the oxidation of the benzyl viologen radical (BV) by nitrite at 546 nm. The solution contained 50 mM potassium phosphate (pH 7.0), 1 mM BV and 10 mM sodium nitrite. BV was reduced by sodium dithionite, and the reaction was started by addition of nitrite. Specific activities were calculated using the protein concentration and an extinction coefficient of ↋_546_ = 19.5 mM^−1^cm^− 1^; 1 unit (U) of enzyme activity is defined as the oxidation of 2 µmol reduced BV min^− 1^ [33, 34, 69]. Alternatively, nitrite reductase activity was determined by following the formation of ammonia by the indophenol reaction; (NH_4_)_2_SO_4_, dried at 80 °C/24 h, was used as standard [70]. All assays were performed in triplicate, with standard deviations less than 10%. Photochemical reduction experiments with 5-deazaflavin/oxalate were carried out according to Massey and Hemmerich [71].

### Ultraviolet/visible and electron paramagnetic resonance spectroscopy

UV/vis spectra were recorded with a Cary 50 spectrometer (Varian) or a Lambda 40 (Perkin Elmer) spectrophotometer; reduced samples were prepared by adding solid sodium dithionite in an anoxic chamber. Difference spectra were recorded by using protein solution without reactant in the reference sample compartment to avoid dilution dependent effects. A home-built sample compartment allowed the use of Thunberg cuvettes to study the reaction of NrfA with NO, hydroxylamine, hydroxylamine derivatives, and sulfite under exclusion of dioxygen.

X-band EPR spectra (perpendicular mode) were recorded on the Bruker Elexsys 500 instrument equipped with the ER 049 X microwave bridge and the 4122 SHQE cavity; spectra (parallel mode) were recorded on the Bruker ESP 300 instrument equipped with the ER 041 MR microwave bridge and the ER 4116 DM bimodal cavity; Suprasil quartz tubes (705-PQ-9.50, Ø_out_ 4 mm, sample volume 250 µl, Wilmad) were used.

### Protein crystallization

The sitting drop vapor diffusion method was used to obtain well-diffracting crystals of CjNrfA and substrate complexes (protein 4.7 mg/mL, temperature 20 °C). Screens were pipetted with the OryxNano protein crystallization robot (Douglas Instruments). Crystallization under anoxic conditions was performed under N_2_/H_2_ at room temperature in an anoxic chamber (Coy Laboratory Products). The crystallization progress was followed either by the Minstrel HT imaging system (Rigaku) or with a microscope. Initial crystals of CjNrfA appeared in 30% PEG 1000 (w/v) and 100 mM CHES/NaOH, pH 9.5. Further crystal optimization was achieved with PEG 1000, range of 35–45% (w/v), 100 mM CHES/NaOH, pH 8.0-9.5 and SrCl_2_ as additive. Best results were obtained by seeding with a freshly prepared seed stock. Crystals of the variant WsNrfA-Lys134His were obtained as described [12, 18].

### Structure determination

Crystals were harvested with a nylon loop and flash-frozen in liquid N_2_; during data collection, crystals were cooled by a gas stream of N_2_ (-173^o^ C) to minimize radiation damage. Diffraction data of CjNrfA and complexes were collected at beamline PXI (Xo6SA) at the Swiss Light Source (Villigen, Switzerland) with a Pilatus 6 M detector at various wavelengths; diffraction data of WsNrfA-Lys134His were obtained in-house using the X-ray generator MicroMax-007 HF (Rigaku) at a wavelength of 1.5418 Å with a Saturn944 + CCD detector (Rigaku). Diffraction data were indexed and integrated with XDS [72] and scaled with AIMLESS [73] of the CCP4 suite [74]. Datasets measured in-house with the Saturn944 + CCD detector were indexed, scaled and integrated with the d*Trek software package [75]. Initial phase information was obtained by the single anomalous dispersion method, with a single dataset measured at the iron edge at 1.739 Å using the autoSHARP suite [76] for automatic phasing and initial model building. Phases of subsequent structures were obtained by molecular replacement with MOLREP [77] as part of the CCP4 suite [74] using the initial model structure as search model. Structure models were refined in iterative steps using REFMAC5 [78] and Coot [79] for model building. Final structures were validated with MolProbity [80]; electrostatic surface potentials were calculated with DelPhi [81], substrate channels with Hollow [82], and visualizations were made with PyMol [83]; Ramachandran statistics were defined by PROCHECK [84]. CjNrfA crystallized in space group P2_1_; WsNrfA-Lys134His crystallized in space group *I* 4_1_22, like wild-type WsNrfA and the WsNrfA-Tyr218Phe variant [12, 20] (Supplementary File, Tables 1 and 2).

## Supplementary Information

Below is the link to the electronic supplementary material.

Supplementary Material 1

## Data Availability

The atomic coordinates and experimental factor amplitudes for the structures of CjNrfA (PDB ID 9TP1, 9TP3, 9TP4) and WsNrfA-Lys134His (PDB ID 32MF) have been deposited with the Protein Data Bank.

## Abbreviations

BN-PAGE: Blue native polyacrylamide gel electrophoresis
CjNrfA: *Campylobacter jejuni *NrfA
EPR: Electron paramagnetic resonance
Deazaflavin: 5-deaza-O-methyl-3-sulfopropyl-isoalloxazine, K^+^ salt
DNRA: Dissimilatory nitrate reduction to ammonium
NrfA: Pentaheme cytochrome *c* nitrite reductase
DdNrfA: *Desulfovibrio desulfuricans* NrfA
DvNrfA: *Desulfovibrio vulgaris* NrfA
EcNrfA: *Escherichia coli* NrfA
GlNrfA: *Geobacter lovleyi* NrfA
GmNrfA: *Geobacter metallireducens* NrfA
SdNrfA: *Sulfurospirillum deleyianum* NrfA
SoNrfA: *Shewanella oneidensis* NrfA
WsNrfA: *Wolinella succinogenes* NrfA
PDB: Protein data bank; http://www.rcsb.org
RMSD: Root mean square deviation
SDS PAGE: Sodium dodecyl sulfate polyacrylamide gel electrophoresis
UV/Vis: Ultraviolet/visible

## References

[1] Simon J (2002) Enzymology and bioenergetics of respiratory nitrite ammonification. FEMS Microbiol Rev 26:285–309. 10.1111/j.1574-6976.2002.tb00616.x 12165429

[2] Kroneck PMH (2022) Nature’s nitrite–to–ammonia expressway, with no stop at dinitrogen. J Biol Inorg Chem 27:1–21. 10.1007/s00775-021-01921-4 34865208 PMC8840924

[3] Hird K, Campeciño JO, Hegg EL (2025) From genes to function: regulation, maturation, and evolution of cytochrome *c* nitrite reductase in nitrate reduction to ammonium. Appl Environ Microbiol e0029225. 10.1128/aem.00292-25 PMC12285233 40488517

[4] Mohan SB, Schmid M, Jetten M, Cole J (2004) Detection and widespread distribution of the nrfA gene encoding nitrite reduction to ammonia, a short circuit in the biological nitrogen cycle that competes with denitrification. FEMS Microbiol Ecol 49:433–443. 10.1016/j.femsec.2004.04.012 19712292

[5] Pandey B, Kumar U, Kaviraj UM, Minick KJ, Mishra AK, Singh JS (2020) DNRA: A short-circuit in biological N-cycling to conserve nitrogen in terrestrial ecosystems. Sci Tot Envir 738:139710. 10.1016/j.scitotenv.2020.139710 32544704

[6] Saghaï A, Pold G, Jones CM, Hallin S (2023) Phyloecology of nitrate ammonifiers and their importance relative to denitrifiers in global terrestrial biomes. Nat Commun 14:8249. 10.1038/s41467-023-44022-3 38086813 PMC10716430

[7] Einsle O, Kroneck PMH (2004) Structural Basis of Denitrification. Biol Chem 385:875–883. 10.1515/BC.2004.115 15551861

[8] Kuypers MMM, Marchant HK, Kartal B (2018) The microbial nitrogen-cycling network. Nat Rev Microbiol 16:263–276. 10.1038/nrmicro.2018.916 29398704

[9] Zhang X, Ward BB, Sigman DM (2020) Global Nitrogen Cycle: Critical Enzymes, Organisms, and Processes for Nitrogen Budgets and Dynamics. Chem Rev 120:5308–5351. 10.1021/acs.chemrev.9b00613 32530264

[10] Pereira IC, Abreu IA, Xavier AV, Le Gall J, Teixeira M (1996) Nitrite reductase from *Desulfovibrio desulfuricans* (ATCC 27774), a heterooligomer heme protein with sulfite reductase activity. Biochem Biophys Res Commun 224:611–618. 10.1006/bbrc.1996.1074 8713097

[11] Stach P, Einsle O, Schumacher W, Kurun E, Kroneck PMH (2000) Bacterial cytochrome *c* nitrite reductase: new structural and functional aspects. J Inorg Biochem 79:381–385. 10.1016/S0162-0134(99)00248-2 10830892

[12] Lukat P, Rudolf M, Stach P, Messerschmidt A, Kroneck PMH, Simon J, Einsle O (2008) Binding and reduction of sulfite by cytochrome *c* nitrite reductase. Biochemistry 47:2080–2086. 10.1021/bi7021415 18201106

[13] Rudolf M, Einsle O, Neese F, Kroneck PMH (2002) Pentahaem cytochrome *c* nitrite reductase: reaction with hydroxylamine, a potential reaction intermediate and substrate. Biochem Soc Trans 30:649–653. 10.1042/bst0300649 12196156

[14] Kemp GL, Clarke TA, Marritt SJ, Lockwood C, Poock SR, Hemmings AM, Richardson DJ, Cheesman MR, Butt JN (2010) Kinetic and thermodynamic resolution of the interactions between sulphite and the pentahaem cytochrome NrfA from *Escherichia coli*. Biochem J 431:73–80. 10.1042/BJ20100866 20629638

[15] Simon J, Kroneck PMH (2013) Microbial Sulfite Respiration. Adv Microb Physiol 62:45–117. 10.1016/B978-0-12-410515-7.00002-0 23481335

[16] Eaves DJ, Grove J, Staudenmann W, James P, Poole RK, White SA, Griffiths L, Cole JA (1998) Involvement of products of the *nrfEFG* genes in the covalent attachment of haem *c* to a novel cysteine–lysine motif in the cytochrome *c*_552_ nitrite reductase from *Escherichia coli*. Mol Microbiol 28:205–216. 10.1046/j.1365-2958.1998.00792.x 9593308

[17] Einsle O, Messerschmidt A, Stach P, Bourenkov GP, Bartunik HD, Huber R, Kroneck PMH (1999) Structure of cytochrome *c* nitrite reductase. Nature 400:476–479. 10.1038/22802 10440380

[18] Einsle O, Stach P, Messerschmidt P, Simon A, Kröger J, Huber A, Kroneck R PMH (2000) Cytochrome *c* nitrite reductase from *Wolinella succinogenes*. Structure at 1.6 Å resolution, inhibitor binding, and heme-packing motifs. J Biol Chem 275:39608–39618. 10.1074/jbc.M006188200 10984487

[19] Clarke TA, Hemmings MA, Burlat B, Butt JN, Cole JA, Richardson DJ (2006) Comparison of the structural and kinetic properties of the cytochrome *c* nitrite reductases from *Escherichia coli*, *Wolinella succinogenes*, *Sulfurospirillum deleyianum* and *Desulfovibrio desulfuricans*. Biochem Soc Trans 34:143–145. 10.1042/BST0340143 16417505

[20] Einsle O (2011) Structure and Function of Formate-Dependent Cytochrome *c* Nitrite Reductase, NrfA. Meth Enzymol 496:399–420. 10.1016/B978-0-12-386489-5.00016-6 21514473

[21] Bamford VA, Angove HC, Seward HE, Thomson AJ, Cole JA, Butt JN, Hemmings AM, Richardson DJ (2002) Structure and Spectroscopy of the Periplasmic Cytochrome *c* Nitrite Reductase from *Escherichia coli*. Biochemistry 41:2921–2931. 10.1021/bi015765d 11863430

[22] Youngblut M, Judd ET, Srajer V, Sayyed B, Goelzer T, Elliott SJ, Schmidt M, Pacheco AA (2012) Laue crystal structure of *Shewanella oneidensis* cytochrome *c* nitrite reductase from a high-yield expression system. J Biol Inorg Chem 17:647–662. 10.1007/s00775-012-0885-0 22382353 PMC3412176

[23] Cunha CA, Macieira S, Dias JM, Almeida G, Goncalves LL, Costa C, Lampreia J, Huber R, Moura JJG, Moura I, Romão MJ (2003) Cytochrome *c* Nitrite Reductase from *Desulfovibrio desulfuricans* ATCC 27774. The relevance of the two calcium sites in the structure of the catalytic subunit (NrfA). J Biol Chem 278:17455–17465. 10.1074/jbc.M211777200 12618432

[24] Rodrigues ML, Oliveira TF, Pereira IAC, Archer M (2006) X-ray structure of the membrane-bound cytochrome *c* quinol dehydrogenase NrfH reveals novel haem coordination. EMBO J 25:5951–5960. 10.1038/sj.emboj.7601439 17139260 PMC1698886

[25] Campeciño J, Lagishetty S, Wawrzak Z, Sosa Alfaro V, Lehnert N, Reguera G, Hu J, Hegg EL (2020) Cytochrome *c* nitrite reductase from the bacterium *Geobacter lovleyi* represents a new NrfA subclass. J Biol Chem 295:11455–11465. 10.1074/jbc.RA120.013981 32518164 PMC7450111

[26] Denkhaus L, Siffert F, Einsle O (2023) An unusual active site architecture in cytochrome *c* nitrite reductase NrfA-1 from *Geobacter metallireducens*. FEMS Microbiol Lett 370:1–8. 10.1093/femsle/fnad068 37460131

[27] Welsh A, Chee-Sanford JC, Connor LM, Löffler FE, Sanford RA (2014) Refined NrfA Phylogeny Improves PCR-Based *nrfA* Gene Detection. Appl Environ Microbiol 80:2110–2119. 10.1128/AEM.03443-13 24463965 PMC3993153

[28] Cannon J, Sanford RA, Connor L, Yang WH, Chee-Sanford J (2019) Sequence alignments and validation of PCR primers used to detect phylogenetically diverse *nrfA* genes associated with dissimilatory nitrate reduction to ammonium (DNRA). Data Brief 25:104016. 10.1016/j.dib.2019.104016 31297410 PMC6596902

[29] Cannon J, Sanford RA, Connor L, Yang WH, Chee-Sanford J (2019) Optimization of PCR primers to detect phylogenetically diverse nrfA genes associated with nitrite ammonification. J Microbiol Methods 160:49–59. 10.1016/j.mimet.2019.03.020 30905502

[30] Parkhill J, Wren BW, Mungall K, Ketley JM, Churcher C, Basham D, Chillingworth T, Davies RM, Feltwell T, Holroyd S, Jagels S, Karlyshev K, Moule AV, Pallen S, Pennk SMJ, Quail CW, Rajandream MA, Rutherford M-A, van Vliet KM, Whitehead AHM, Barrell S BG (2000) The genome sequence of the food-borne pathogen *Campylobacter jejuni* reveals hypervariable sequences. Nature 403:665–668. 10.1038/35001088 10688204

[31] Taylor AJ, Kelly DJ (2019) The function, biogenesis and regulation of the electron transport chains in *Campylobacter jejuni*: New insights into the bioenergetics of a major food-borne pathogen. Adv Microb Physiol 74:239–329. 10.1016/bs.ampbs.2019.02.003 31126532

[32] Mondry Cohen N, Krishna Kumar C, Iitoyo H, Rookyard AW, Cain JA, Man L, White MY, Dale AL, Cordwell SJ (2025) Exploring the Targets of Reactive Oxygen Species and Defense against Oxidative Stress in *Campylobacter jejuni* Using a Multiomics Approach. J Proteome Res 24:3032–3048. 10.1021/acs.jproteome.5c00182 40426317

[33] Pisa R, Stein T, Eichler R, Gross R, Simon J (2002) The *nrfI* gene is essential for the attachment of the active site haem group of *Wolinella succinogenes* cytochrome *c* nitrite reductase. Mol Microbiol 43:763–770. 10.1046/j.1365-2958.2002.02784.x 11929530

[34] Kern M, Eisel F, Scheithauer J, Kranz RG, Simon J (2010) Substrate specificity of three cytochrome *c* haem lyase isoenzymes from *Wolinella succinogenes*: unconventional haem *c* binding motifs are not sufficient for haem *c* attachment by NrfI and CcsA1. Mol Microbiol 75:122–137. 10.1111/j.1365-2958.2009.06965.x 19919672 PMC3414424

[35] Kern M, Simon J (2011) Production of recombinant multiheme cytochromes *c* in *Wolinella succinogenes*. Methods Enzymol 486: 429 – 46. 10.1016/B978-0-12-381294-0.00019-5. PMID: 21185447 21185447

[36] Brittain T, Blackmore R, Greenwood C, Thomson A (1992) Bacterial nitrite-reducing enzymes. Eur J Biochem 209:793–802. 10.1111/j.1432-1033.1992.tb17350.x 1425687

[37] Schumacher W, Hole U, Kroneck PMH (1994) Ammonia-forming cytochrome *c* nitrite reductase from *Sulfurospirillum deleyianum* is a tetraheme protein: new aspects of the molecular composition and spectroscopic properties. Biochem Biophys Res Commun 205:911–916. 10.1006/bbrc.1994.2751 7999130

[38] Einsle O, Schumacher W, Kurun E, Nath U, Kroneck PMH (1998) Cytochrome *c* Nitrite Reductase from *Sulfurospirillum Deleyianum* and *Wolinella Succinogenes*. In: Canters GW, Vijgenboom E (eds) Biological Electron Transfer Chains: Genetics, Composition and Mode of Operation. NATO ASI Series, vol 512. Springer, Dordrecht. 10.1007/978-94-011-5133-7_14.

[39] Bykov D, Neese F (2011) Substrate binding and activation in the active site of cytochrome *c* nitrite reductase: a density functional study. J Biol Inorg Chem 16:417–430. 10.1007/s00775-010-0739-6 21125303

[40] Pittman MS, Elvers KT, Lee L, Jones MA, Poole RK, Park SF, Kelly DJ (2007) Growth of *Campylobacter jejuni* on nitrate and nitrite: electron transport to NapA and NrfA via NrfH and distinct roles for NrfA and the globin Cgb in protection against nitrosative stress. Mol Microbiol 63:575–590. 10.1111/j.1365-2958.2006.05532.x 17241202

[41] Mowat C, Rothery E, Miles C, McIver L, Doherty MK, Drewette K, Taylor P, Walkinshaw MD, Chapman SK, Reid GA (2004) Octaheme tetrathionate reductase is a respiratory enzyme with novel heme ligation. Nat Struct Mol Biol 11:1023–1024. 10.1038/nsmb827 15361860

[42] Einsle O, Messerschmidt A, Huber R, Kroneck PMH, Neese F (2002) Mechanism of the Six-Electron Reduction of Nitrite to Ammonia by Cytochrome *c* Nitrite Reductase. J Am Chem Soc 124:11737–11745. 10.1021/ja0206487 12296741

[43] Bykov D, Neese F (2012) Reductive activation of the heme iron–nitrosyl intermediate in the reaction mechanism of cytochrome *c* nitrite reductase: a theoretical study. J Biol Inorg Chem 17:741–760. 10.1007/s00775-012-0893-0 22454108

[44] Bykov D, Plog M, Neese F (2014) Heme-bound nitroxyl, hydroxylamine, and ammonia ligands as intermediates in the reaction cycle of cytochrome *c* nitrite reductase: a theoretical study. J Biol Inorg Chem 19:97–112. 10.1007/s00775-013-1065-6 24271207

[45] Bykov D, Neese F (2015) Six-Electron Reduction of Nitrite to Ammonia by Cytochrome *c* Nitrite Reductase: Insights from Density Functional Theory Studies. Inorg Chem 54:9303–9316. 10.1021/acs.inorgchem.5b01506 26237518

[46] Zhong F, Alden SL, Hughes RP, Pletneva EV (2022) Comparing Properties of Common Bioinorganic Ligands with Switchable Variants of Cytochrome *c*. Inorg Chem 61:1207–1227. 10.1021/acs.inorgchem.1c02322 34699724

[47] Deng Y, Carnevale V, Ditchfield R, Pletneva EV (2024) Applications of the Newly Developed Force-Field Parameters Uncover a Dynamic Nature of Ω–Loop C in the Lys-Ligated Alkaline Form of Cytochrome *c*. J Phys Chem B 128:5935–5949. 10.1021/acs.jpcb.4c00625 38864552

[48] Johnson EA, Rice SL, Preimesberger MR, Nye DB, Gilevicius L, Wenke BB, Brown JM, Witman GB, Lecomte JTJ (2014) Characterization of THB1, a *Chlamydomonas reinhardtii* truncated hemoglobin: linkage to nitrogen metabolism and identification of lysine as the distal heme ligand. Biochemistry 53:4573–4589. 10.1021/bi5005206 24964018 PMC4108185

[49] Tikhonova TV, Slutsky A, Antipov AN, Boyko KM, Polyakov KM, Sorokin DY, Zvyagilskaya RA, Popov VO (2006) Molecular and catalytic properties of a novel cytochrome *c* nitrite reductase from nitrate-reducing haloalkaliphilic sulfur-oxidizing bacterium *Thioalkalivibrio nitratireducens*. Biochim Biophys Acta 1764:715–723. 10.1016/j.bbapap.2005.12.021 16500161

[50] Du J, Perera R, Dawson JH (2011) Alkylamine-ligated H93G myoglobin cavity mutant: a model system for endogenous lysine and terminal amine ligation in heme proteins such as nitrite reductase and cytochrome *f*. Inorg Chem 50:1242–1249. 10.1021/ic101644u 21250678 PMC3047591

[51] Du J, Sono M, Dawson JH (2011) The H93G Myoglobin Cavity Mutant as a Versatile Scaffold for Modeling Heme Iron Coordination Structures in Protein Active Sites and Their Characterization with Magnetic Circular Dichroism Spectroscopy. Coord Chem Rev 255:700–716. 10.1016/j.ccr.2011.01.029 21423881 PMC3060032

[52] Tse W, Whitmore N, Cheesman MR, Watmough NJ (2021) Influence of the heme distal pocket on nitrite binding orientation and reactivity in Sperm Whale myoglobin. Biochem J 478:927–942. 10.1042/BCJ20200596 33543749 PMC7925009

[53] Soares R, Costa NL, Paquete CM, Andreini C, Louro RO (2022) A New Paradigm of Multiheme Cytochrome Evolution by Grafting and Pruning Protein Modules. Mol Biol Evol 39:msac139. 10.1093/molbev/msac139 35714268 PMC9250108

[54] Saghaï A, Hallin S (2024) Diversity and ecology of NrfA-dependent ammonifying microorganisms. Trends Microbiol 32:602–613. 10.1016/j.tim.2024.02.007 38462391

[55] Chivers PT, Basak P, Maroney MJ (2024)One His, two His… the emerging roles of histidine in cellular nickel trafficking.J Inorg Biochem 259: 112668, 10.1016/j.jinorgbio.2024.112668 39053077

[56] Clarke TA, Edwards MJ, Gates AJ, Hall A, White GF, Bradley J, Reardon CL, Shi L, Beliaev AS, Marshall MJ, Wang Z, Watmough NJ, Fredrickson JK, Zachara JM, Butt JN, Richardson DJ (2011) Structure of a bacterial cell surface decaheme electron conduit. Proc Natl Acad Sci USA 108:9384–9389. 10.1073/pnas.1017200108 21606337 PMC3111324

[57] Breuer M, Rosso KM, Blumberger J (2014) Electron flow in multiheme bacterial cytochromes is a balancing act between heme electronic interaction and redox potentials. Proc Natl Acad Sci USA 111:611–616. 10.1073/pnas.1316156111 24385579 PMC3896160

[58] Milton RD, Minteer SD (2017) Enzymatic Bioelectrosynthetic Ammonia Production: Recent Electrochemistry of Nitrogenase, Nitrate Reductase, and Nitrite Reductase. ChemPlusChem 82:513–521. 10.1002/cplu.201600442 31961593

[59] Akram M, Dietl A, Mersdorf U, Prinz S, Maalcke WJ, Keltjens JT, Ferousi C, de Almeida NM, Reimann J, Kartal B, Jetten MSM, Parey K, Barends TRM (2019) A 192-heme electron transfer network in the hydrazine dehydrogenase complex. Sci Adv 5:eaav4310. 10.1126/sciadv.aav4310 31001586 PMC6469936

[60] Edwards MJ, White GF, Butt JN, Richardson DJ, Clarke TA (2020) The Crystal Structure of a Biological Insulated Transmembrane Molecular Wire. Cell 181:665–673. 10.1016/j.cell.2020.03.032 32289252 PMC7198977

[61] Trindade IB, Paquete CM, Louro RO (2021) Extracellular redox chemistry. Met Ions Life Sci 21:229–269. 10.1515/9783110589771-008

[62] Futera Z, Ide I, Kayser B, Garg K, Jiang X, van Wonderen JH, Butt JN, Ishii I, Pecht I, Sheves M, Cahen D, Blumberger J (2020) Coherent Electron Transport across a 3 nm Bioelectronic Junction Made of Multi-Heme Proteins. J Phys Chem Lett 11:9766–9774. 10.1021/acs.jpclett.0c02686 33142062 PMC7681787

[63] Piper SEH, Casadevall C, Reisner E, Clarke TA, Jeuken LJC, Gates AJ, Butt JN (2022) Photocatalytic Removal of the Greenhouse Gas Nitrous Oxide by Liposomal Microreactors. Angew Chem Int Ed 61:e202210572. 10.1002/anie.202210572 PMC9825952 35951464

[64] Dong M, Nielsen LP, Yang S, Klausen LH, Xu M (2024) Cable bacteria: widespread filamentous electroactive microorganisms protecting environments. Trends Microbiol 32:697–706. 10.1016/j.tim.2023.12.001 38151387

[65] Costa C, Moura JJG, Moura I, Wang Y, Huynh BH (1996) Redox Properties of Cytochrome c Nitrite Reductase from *Desulfovibrio desulfuricans* ATCC 27774. J Biol Chem 271:23191–23196. 10.1074/jbc.271.38.23191 8798514

[66] Almeida MG, Macieira S, Goncalves LL, Huber R, Cunha CA, Romão MJ, Costa C, Lampreia J, Moura JJG, Moura I (2003) The isolation and characterization of cytochrome c nitrite reductase subunits (NrfA and NrfH) from *Desulfovibrio desulfuricans* ATCC 27774. Re-evaluation of the spectroscopic data and redox properties. Eur J Biochem 270:3904–3915. 10.1046/j.1432-1033.2003.03772.x 14511372

[67] Walker FA (2004) Models of the Bis-Histidine-Ligated Electron-Transferring Cytochromes. Comparative Geometric and Electronic Structure of Low-Spin Ferro- and Ferrihemes. Chem Rev 104:589–616. 10.1021/cr020634 14871136

[68] Zoppellaro G, Bren KL, Ensign AA, Harbitz E, Kaur R, Hersleth H-P, Ryde U, Hederstedt L, Andersson KK (2009) Studies of Ferric Heme Proteins with Highly Anisotropic/Highly Axial Low Spin (S = 1/2) Electron Paramagnetic Resonance Signals with bis-Histidine and Histidine-Methionine Axial Iron Coordination. Biopolymers 91:1064–1082. 10.1002/bip.21267 19536822 PMC2852197

[69] Bokranz M, Katz J, Schröder I, Roberton AM, Kröger A (1983) Energy metabolism and biosynthesis of *Vibrio succinogenes* growing with nitrate or nitrite as terminal electron acceptor. Arch Microbiol 135:36–41. 10.1007/BF00419479

[70] Boltz DF, Taras MJ (1978) Nitrogen. In Colorimetric determination of nonmetals. Chemical analysis. Edited by Boltz, D.F., Howell, J.A., Vol. 8, pp. 197–251. John Wiley & Sons Inc., New York, Chichester, Brisbane, Toronto

[71] Massey V, Hemmerich P (1977) A photochemical procedure for reduction of oxidation-reduction proteins employing deazariboflavin as catalyst. J Biol Chem 252:5612–5614 407227

[72] Kabsch W (2010) XDS. Acta Cryst D 66:125–132. 10.1107/S0907444909047337 20124692 PMC2815665

[73] Evans P (2006) Scaling and assessment of data quality. Acta Cryst D 62:72–82. 10.1107/S0907444905036693 16369096

[74] Winn MD, Ballard CC, Cowtan KD, Dodson EJ, Emsley P, Evans PR, Keegan RM, Krissinel EB, Leslie AG, W, McCoy A, McNicholas SJ, Murshudov GN, Pannu NS, Potterton EA, Powell HR, Read RJ, Vagin AKS (2011) Overview of the CCP4 suite and current developments. Acta Cryst D 67:235–242. 10.1107/S0907444910045749 21460441 PMC3069738

[75] Pflugrath JW (1999) The finer things in X-ray diffraction data collection. Acta Cryst D 55:1718–1725. 10.1107/S090744499900935X 10531521

[76] Vonrhein C, Blanc E, Roversi P, Bricogne G (2007) Automated Structure Solution With autoSHARP. In: Doublié, S. (eds) Macromolecular Crystallography Protocols. Methods in Molecular Biology™, 364: 215–230, Humana Press. 10.1385/1-59745-266-1:215 17172768

[77] Vagin A, Teplyakov A (1997) MOLREP: an automated program for molecular replacement. J Appl Cryst 30:1022–1025. 10.1107/S0021889897006766

[78] Murshudov GN, Skubák P, Lebedev AA, Pannu NS, Steiner RA, Nicholls RA, Winn MD, Long F, Vagin AA (2011) REFMAC5 for the refinement of macromolecular crystal structures. Acta Cryst D 67:355–367. 10.1107/S0907444911001314 21460454 PMC3069751

[79] Emsley P, Cowtan K (2004) COOT: model-building tool for molecular graphics. Acta Cryst D 60:2126–2132. 10.1107/S0907444904019158 15572765

[80] Chen VB, Arendall IIIWB, Headd JJ, Keedy DA, Immormino RM, Kapral GJ, Murray LW, Richardson JS, Richardson DC (2010) MolProbity: all-atom structure validation for macromolecular crystallography. Acta Cryst D 66:12–21. 10.1107/S0907444909042073 20057044 PMC2803126

[81] Honig B, Nicholls A (1995) Classical electrostatics in biology and chemistry. Science 268:1144–1149. 10.1126/science.7761829 7761829

[82] Ho B, Gruswitz F (2008) HOLLOW: Generating accurate representations of channel and interior surfaces in molecular structures. BMC Struct Biol 8:49. 10.1186/1472-6807-8-49 19014592 PMC2603037

[83] DeLano WL (2002) The PyMOL Molecular Graphics System. https://ci.nii.ac.jp/naid/10020095229

[84] Laskowski RA, MacArthur MW, Moss DS, Thornton JM (1993) PROCHECK: a program to check the stereochemical quality of protein structures. J Appl Cryst 26:283–291. 10.1107/S0021889892009944

