# Supplementary material for: Lysine or histidine? examining the role of the proximal ligand at the active heme center of cytochrome c nitrite reductase
Source: J Biol Inorg Chem. 2026 Jul 28;31(5):317–28. doi: 10.1007/s00775-026-02165-w (PMC13562315; doi:10.1007/s00775-026-02165-w)
Supplement: Supplementary file 1 [file 775_2026_2165_MOESM1_ESM.docx]

**Supplementary File**

**Fig. 1** Size exclusion chromatography (Superdex S 200) and SDS PAGE (Coomassie Blue staining) of wild-type WsNrfA (**A,B**) and variant WsNrfA-Lys134His (**D,E**). Both proteins eluted at a retention volume corresponding to a dimer, 110 kDa; peak (1) and tail (2) fractions showed each one band at 58 kDa corresponding to a monomer; MW, marker proteins.

**Fig. 2** Size exclusion chromatography (Superdex S 200) (**A**), SDS PAGE (**B**), and BN PAGE (**C**) of CjNrfA. The protein eluted at a retention volume corresponding to a dimer, 176 kDa; pooled fractions (shaded) were concentrated to 12.3 mg/mL; SDS-PAGE of concentrated protein, lane 1 (Coomassie staining), lane 2 (heme staining); lower band corresponds to a monomer, 73 kDa, the upper band to a tetramer, 280 kDa; BN PAGE of the concentrated protein shows one band at 280 kDa corresponding to a tetramer; MW, marker proteins.

**Fig. 3** Structure of the CjNrfA active site cavity (**A, B**), with nitrite (**C**) or sulfite (**D**) bound to the distal side of heme-1 iron. His154 provides the proximal ligand); Arg129, Tyr265 and His366 form the catalytic triad. Omit maps for NO_2_^-^ and SO_3_^2-^ are contoured at a σ-level of 3.0. (**A**) H_2_O occupies the substrate binding site (**B**) Ca^2+^ I resides above heme-1, distance 10.4 Å (**C**) NO_2_^-^ is N-bound, ligated by Arg129 and His366 (**D**) SO_3_^2-^ is S-bound, ligated by Arg129, Tyr265 and His366.

**Fig. 4** Cross-sections through the molecular surface of NrfA enzymes, emphasizing the substrate channel structure and the substrate (S) and product (P) pathways. (**A**) WsNrfA (PDB 1FS7) has one channel (**B**) SdNrfA (PDB 1QDB) the channel splits into two exit channels (**C**) DvNrfA (PDB 2J7A) the exit channel is blocked by an additional loop (**D**) CjNrfA, C-terminal helix α 27 blocks the exit channel.

**Table 1**. Data collection and refinement statistics of native CjNrfA, and its nitrite and sulfite complexes

| Data Set |  | native | NO_2_^-^ soak | SO_3_^2-^ soak |
| --- | --- | --- | --- | --- |
| space group |  | *P*2_1_ | *P*2_1_ | *P*2_1_ |
| cell constants *a*, *b*, *c* [Å]  α, β, γ [°] |  | 64.7, 159.0, 68.0  90.0, 117.2, 90.0 | 64.5, 160.8, 65.3  90.0, 118.1, 90.0 | 64.6, 160.8, 68.6  90.0, 116.5, 90.0 |
| resolution limits [Å] |  | 48.14 – 1.60 (1.63 – 1.6) | 57.60 – 2.29 (2.37 – 2.29) | 48.79 – 1.90 (1.93 – 1.90) |
| completeness (%) |  | 96.8 (96.3) | 93.7 (86.4) | 99.6 (99.8) |
| unique reflections |  | 154,998 (7,608) | 49,340 | 97,753 (4,777) |
| multiplicity (%) |  | 2.3 (2.4) | 3.0 (3.0) | 6.6 (6.7) |
| *R*_merge_ ^a)^ |  | 0.098 (0.545) | 0.185 (0.442) | 0.110 (0.607) |
| *R*_p.i.m.[__12__]_ |  | 0.082 (0.446) | - | 0.046 (0.253) |
| mean I/σ(I) |  | 8.6 (2.1) | 3.8 (2.0) | 11.6 (3.3) |
| CC_1/2[__13__]_ |  | 0.983 (0.491) | - | 0.997 (0.860) |
| refinement statistics | | | |  |
| *R*_cryst_^b)^ |  | 0.21 | 0.22 | 0.18 |
| *R*_free_ (%) |  | 0.23 | 0.25 | 0.19 |
| non-hydrogen atoms |  | 10,534 | 9,712 | 10,014 |
| solvent molecules |  | 1,076 | 300 | 549 |
| Cruickshank’s DPI [14] |  | 0.109 | 0.583 | 0.125 |
| figure of merit |  | 0.816 | 0.787 | 0.873 |
| r.m.s. deviations from ideal values | | | |  |
| bond lengths (Å) |  | 0.015 | 0.0079 | 0.0069 |
| bond angles (º) |  | 1.033 | 1.330 | 1.116 |
| average *B* values (Å^2^) | | | |  |
| protein main chain atoms |  | 15.55 | 38.40 | 24.95 |
| protein all atoms |  | 15.74 | 39.02 | 25.43 |
| solvent |  | 24.91 | 32.65 | 29.63 |
| ligand |  |  | 42.07 | 28.13 |
| ^a^ *R*_merge_ = Σ*_hkl_* [(Σ*_i_* \|*I_i_* - ‹*I*›\|) / Σ*_i_ I_i_*]; ^b^ *R*_cryst_ = Σ*_hkl_* \|\|*F*_obs_\| - \|*F*_calc_\|\| / Σ*_hkl_* \|*F*_obs_\|; *R*_free_ is the cross-validation *R* value for a test set of 5 % of unique reflections; ^c^ Ramachandran statistics | | | |  |

**Table 2.** Data collection and refinement statistics of WsNrfA-Lys134His variant

Data Set	WsNrfA-Lys134His

Space group	*I* 4_1_22

Unit cell dimensions	a = 119.97 Å, b = 119.97 Å, c = 186.24 Å

α = 90 °, ß = 90 °, γ = 90 °

Resolution (Å)	24.81 – 2.05

No. of reflections

All	42.984

Observed	38.514

Completeness (%)

Total	89.6

Last shell (2.16-2.05 Å)	32.3

R_merge_^a^ (%)

Total	9.8

Last shell (2.16-2.05 Å)	54.0

I/σ(I)

Total	6.8

Last shell (2.16-2.05 Å)	1.4

Redundancy

Total	5.3

Last shell (2.16-2.05 Å)	1.5

Data used in refinement

Resolution range (Å)	24.82 – 2.20

Data cutoff σ(F)	0.0

Number of reflections (all)	34.812

Number of reflections (observed)	34.673

Completeness (working + test set) (%)	99.6

R-value (working set)	0.178

Free R-value (test set, 9.9%)	0.231

Fit in highest resolution bin

Resolution range (Å)	2.34 – 2.20

Bin completeness (working + test set) (%)	98.9

Bin R-value	0.263

Bin Free-R-value	0.303

No. of atoms

Protein	3773

5 Heme groups	215

Ca^2+^	1

Y^3+^	3

Water	587

2 Acetate	8

1 Monochloroacetate	5

Estimated coordinate error from

Luzzati plot (Å)	0.22

r.m.s. Deviation

Bond length (Å)	0.006

Bond angles (°)	1.1

Dihedral angles (°)	21.3

Improper angles (°)	1.2

B-factors (Å^2^)

All atoms	33.8

Protein atoms	32.3

Main chain atoms	30.8

Side chain atoms	33.9

heme cofactors	28.0

Ca^2+^	27.1

Y^3+^	43.3

Acetate	38.0

Monochloroacetate	28.5

Water atoms	44.6

r.m.s. bonded B’s	3.6
